# Geographical divides in male premature mortality in the CEE–FSU European region: an ecological study of 2320 spatial units in 12 countries, 2003–2019

**DOI:** 10.1136/bmjph-2025-003714

**Published:** 2026-02-20

**Authors:** Pavel Grigoriev, Domantas Jasilionis, Sergey Timonin, Nataliia Levchuk, Pavlo Shevchuk, Olga Penina, Katalin Kovács, Sebastian Klüsener

**Affiliations:** 1 Federal Institute for Population Research (BiB), Wiesbaden, Germany; 2 Max-Planck-Institute for Demographic Research, Rostock, Germany; 3 Vytautas Kavolis Transdisciplinary Research Institute, Vytautas Magnus University, Kaunas, Lithuania; 4 School of Demography, The Australian National University, Canberra, Australian Capital Territory, Australia; 5 National Center for Epidemiology and Population Health, The Australian National University, Canberra, Australian Capital Territory, Australia; 6 Mykhailo Ptukha Institute for Demography and Life Quality Research, Kyiv, Ukraine; 7 Nicolae Testemitanu State University of Medicine and Pharmacy, Chisinau, Moldova; 8 Hungarian Demographic Research Institute, Budapest, Hungary; 9 Department of Sociology and Social Psychology (DSS), University of Cologne, Cologne, Germany

**Keywords:** Public Health, Epidemiology, Community Health, Demography

## Abstract

**Introduction:**

Male premature mortality has been one of the most critical components of the persistent health disadvantage observed in the countries of the former Soviet Union (FSU) and Central-Eastern Europe (CEE). The spatial dimension of this phenomenon has remained largely unexplored.

**Methods:**

We created a unique collection of harmonised regional cause-specific mortality data to assess spatiotemporal patterns of male premature mortality across 2320 territorial units in 12 FSU and CEE countries during 2003–2019. As an indicator of premature mortality, we apply the age-standardised mortality rate for the age group 20–64 years. Choropleth maps are employed to visualise the spatial patterns for all-cause and cause-specific mortality as well as statistically significant *hot* and *cold* spots, identified with the Getis-Ord Gi* statistic.

**Results:**

Between 2003 and 2019, there was a substantial reduction in mortality levels across all countries and spatial units as well as a slight reduction in regional inequality. The most pronounced mortality disparities are visible within the FSU block: between regions of Russia and the Baltic States as well as between the neighbouring regions of Belarus and Ukraine. Several countries exhibit spatial gradients that are occasionally extending into neighbouring countries. The most notable gradients include a North-West–South gradient in Russia and East-West mortality divides in Hungary, Romania and Poland.

**Conclusion:**

Despite notable political and socioeconomic differences between the former socialist countries, the spatial mortality patterns have remained remarkably persistent across and within them. Historical and sociocultural contexts should be considered while interpreting contemporary mortality patterns.

**WHAT IS ALREADY KNOWN ON THIS TOPIC**

- Male premature mortality remains one of the most critical components of the persistent health disadvantage observed in the countries of the former Soviet Union (FSU) and Central-Eastern Europe (CEE).
- Most existing studies focus on differences between countries, while the detailed spatial dimension of this phenomenon remains largely underexplored.

**WHAT THIS STUDY ADDS**

- For the first time, we created a unique collection of harmonised cause-specific mortality data at high time-constant geographical detail to explore spatiotemporal patterns of male premature mortality across small territorial units in 12 FSU and CEE countries.
- Our analyses suggest the high relevance of historical and sociocultural contexts for interpreting contemporary spatial patterns. These patterns have remained remarkably persistent across and within countries, despite new geopolitical divides and differences in socioeconomic development.

**HOW THIS STUDY MIGHT AFFECT RESEARCH, PRACTICE OR POLICY**

- Tracing geographical patterns and focusing on cross-border continuities is relevant in the context of geopolitical divisions between the new European Union member states and other FSU countries, such as Belarus, Moldova, Russia and Ukraine.

## Introduction

 In Europe, the persistent East–West life expectancy divide still remains one of the key public health challenges for achieving more sustainable and equitable health. Despite some significant improvements during the most recent decades, progress in reducing excess mortality in the countries of Central-Eastern Europe (CEE) and the former Soviet Union (FSU) remains highly uneven across countries and over time. Notable disparities in life expectancy improvements can be observed in the CEE countries and the Baltic States, which all benefited from EU membership since 2004 or 2007.^1^ Between the mid-2000s and 2019, also substantial (although more varying) health progress occurred in the initially worst-performing FSU countries, such as Russia, Belarus, Ukraine and Moldova.^2^,^6^ Except for a few positive examples, including Czechia and Poland, the majority of CEE countries still do not show any systematic convergence towards life expectancy levels observed in Western Europe.^1^ The striking scale of mortality divergence across CEE–FSU countries becomes even more evident when exploring patterns at the subnational level.^7^,^10^

One of the most critical components of the persisting health crisis in the CEE–FSU region remains male premature mortality driven by mortality from cardiovascular diseases (CVDs) and external causes of death.^11^ This specific mortality pattern, originating from the era of communist rule or even from a more distant past, is often associated with prevailing harmful alcohol consumption cultures and alcohol-related violent deaths.^7 12^ By tracing spatial disparities of excess premature mortality across small territorial units in 12 CEE and FSU countries, this study aims to provide new and more detailed perspectives on common and country-specific determinants of this persisting public health challenge in Europe. Considering the unique historical contexts characterised by numerous shifts of political borders and the legacies from the period of communist rule, we assess spatial disparities of male premature mortality and its changes over time in pre-pandemic CEE–FSU region with a special focus on cross-border regions.

Focusing on cross-border regions offers a fascinating context for investigating reasons for observed mortality differentials. Despite sharing cultural and historical similarities, populations on either side of a border operate within distinct institutional environments. Thus, cross-border regions can be designated as natural *laboratories* for evaluating the impact of varying health policies, individual health behaviour and healthcare systems on mortality.^13^ Despite its promise, this area of research has received limited scholarly attention.

## Data and methods

### Data collection and harmonisation

Our analyses are based on official regional mortality data originating from the selected FSU (Belarus, Ukraine, Russia (European part), Moldova, Latvia, Lithuania, Estonia) and CEE countries (Poland, Slovakia, Czechia, Hungary, Romania). The separation between CEE and FSU countries reflects key differences between these geopolitical country blocks. In the past, CEE countries were sovereign socialist states with stronger Western ties and a smoother transition to market economy. In contrast, FSU countries were integral parts of the Soviet Union, inherited deeper Soviet institutional legacies, experienced more turbulent transitions accompanied by markedly different mortality trends. The obtained raw data include death counts by year, sex, 5 year age group, cause of death and district over the period 2003–2019. To make the data comparable over time, we harmonised them to conform to the most recent territorial division. The shape files were modified accordingly. Our final dataset consists of 2320 spatial units (districts and cities), of which 1723 are located in FSU and 597 in CEE countries (see online supplemental table S1 and figure S1). We focus on all-cause male premature mortality and its main drivers: cardiovascular (items I00–I99, G45 in the ICD–10), and external (V01–Y98) mortality. To ensure better data comparability, we applied the proportional redistribution of ill-defined causes of death (R00–R53, R55–R94, R96–R99) for each spatial unit, period and 5 year age group.

In this study, we define male premature mortality as mortality occurring at ages 20 to 64 years. This definition is consistent with numerous previous studies in the region,^14^,^17^ which identified mortality at working ages as the main driver of overall male mortality trends. We focus on spatial mortality patterns in CEE and FSU countries, and their changes between 2003–2005 and 2017–2019. Before computing mortality rates, death and population counts were pooled over these 3 year periods to ensure more robust estimates. The choice of these time periods was driven by several considerations: (a) Data availability and comparability of cause-specific mortality data (the transition to ICD–10 was completed by 2003 in all countries except Ukraine), (b) Proximity to the population censuses in the countries for which reliable intercensal population estimates are either not available or not reliable (Moldova and Russia), (c) 2003–2005 as a turning point in mortality dynamics in many FSU countries and (d) 2017–2019 as the last years before the COVID-19 pandemic.

We made substantial efforts to harmonise the data. However, a few noteworthy limitations remain. For Latvia, regional cause-specific mortality data were not available. For Russia, we have to use for the first observation period the years 2001–2003 instead of 2003–2005 so that the 2002 population census can be used as the denominator and the midpoint. Likewise, mortality rates for Moldova are centred around the 2004 population census and refer to the 5 year period 2002–2006. Finally, the initial point of the analysis for Ukraine refers to the period 2006–2008 due to data availability constraints as well as the late transition to the ICD–10 in 2005. Keeping these data comparability issues in mind, these slightly deviating periods can still be used as a reasonable approximation for the initial and the last points of our analyses. Thus, we refer to them as such (2003–2005 and 2017–2019) thereafter. The COVID-19 pandemic years are not covered by our analyses because of data availability constraints for the major FSU countries: Belarus, Russia and Ukraine. Because of Russia’s annexation of Crimea and the war in Donbas since 2014, our analyses do not cover Crimea, Donetsk and Luhansk oblasts of Ukraine.

### Methods

To compute age-cause-specific mortality rates, we either rely on official data for the mid-year population or estimated population exposures (denominator) as the mean value of the population size observed at the beginning and the end of a calendar year. For Russia and Moldova, we employ data from the population censuses. For Lithuania, deaths for the period 2003–2005 were derived from unpublished aggregated census-linked database provided by Statistics Lithuania, whereas deaths for 2017–2019 and population denominators for both periods were downloaded from the online databases by the Institute of Hygiene and Statistics Lithuania, respectively. As a mortality indicator, we apply the age-standardised mortality rate (SDR) calculated using the direct method of standardisation and the European population standard (1976). Using this old standard is more appropriate for assessing long-term mortality as well as making correct comparisons with previous studies based on the same standard. As a measure of inequality, we employ the Theil Index,^18^ which allows us to assess the degree of heterogeneity across all spatial units and within each country block, as well as to measure trends in spatial inequalities, and to decompose them into *within-country* and *between-country* components (see online supplemental A for the detailed description).

All spatial data operations, as well as all spatial analyses, are performed using *ArcGIS* software. The maps of male premature mortality are categorised using *quantile* classification. To identify statistically significant *hot* and *cold* spots of premature mortality, we rely on the Getis-Ord Gi* statistic^19^ and the *first-order queen* conceptualisation of spatial relationships. The latter implies that spatial units that share at least one common border point are considered neighbours. Statistically significant hot spots (clusters of high values) and cold spots (clusters of low values) are detected using the Hot Spot Analysis tool in ArcGIS. This tool calculates z-scores and p values for each feature, categorising them based on these values. A high positive z-score and a small p value suggest the spatial clustering of high values (hot spot), while a low negative z-score and a small p value indicate the clustering of low values (cold spot). Larger absolute z-scores signify stronger clustering, whereas a z-score close to zero indicates the absence of noticeable spatial clustering.

### Patient and public involvement

None.

## Results

### Long-term mortality trends

**Figure 1:**
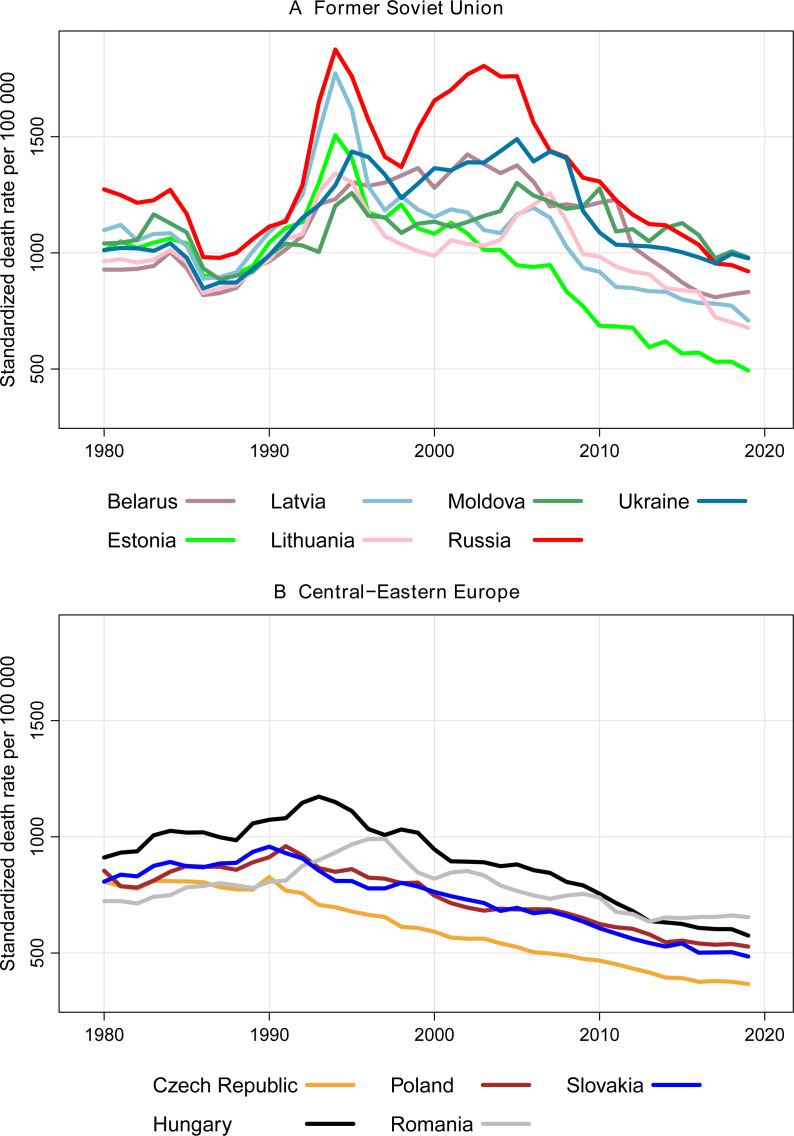
Trends in male premature mortality in 12 former Soviet Union (A) and Central-Eastern European (B) countries, 1980–2019. Source: own calculations based on the data from the Human Mortality Database (HMD) and official statistics. Note: since 2014, data on Ukraine do not include Crimea, Donetsk and Luhansk oblasts.

Long-term trends in male premature mortality in the FSU and the CEE countries are shown in figure 1. Already in the beginning of the 1980s, significant differences were observed between two groups of countries, with Russia demonstrating the highest mortality rates.

Within the FSU group (Panel A of figure 1), all countries experienced simultaneous abrupt declines in mortality in the mid-1980s attributable to Gorbachev’s anti-alcohol campaign. The short-term improvement in mortality was followed by a subsequent mortality increase in the late 1980s. The acute mortality crisis of the early 1990s was triggered by the collapse of the Soviet Union and a subsequent deep socioeconomic crisis.^20^ The increases in male premature mortality were particularly pronounced in Russia and Latvia. In 1994, the SDR reached its maximum of 1875 and 1773 deaths per 100 000 population in Russia and Latvia, respectively. In 1990, the corresponding figures were almost twice as low and constituted 1113 and 1090 deaths per 100 000, respectively. The late 1990s marked an important turning point where mortality trends began to diverge substantially not only within the FSU block but also among the Baltic States. There was another huge increase in premature mortality in Russia caused by the financial crisis of 1998.^17^ Mortality trends in the other FSU countries were less affected by this crisis. Ukraine, Moldova and Lithuania witnessed only modest mortality increases in this period. Meanwhile, there was a stagnation in Belarus, a modest improvement in Latvia and a steady reduction in Estonia. Starting from the late 2000s, all FSU countries joined the trajectory of a steady mortality decline. By the end of the observation period, the highest male premature mortality level is evident in Moldova, Ukraine and Russia, with almost no differences between these countries. These are followed by Belarus, Latvia and Lithuania, which exhibit lower levels. The lowest mortality level close to the best-performing countries of the CEE block is observed in Estonia as the vanguard country of the region.

Compared with the FSU countries, mortality trends in the CEE countries (Panel B of figure 1) were not that turbulent. Historically, apart from Hungary, male premature mortality in these countries was substantially lower. Within our observation period, only the short-term period of mortality improvements in the FSU in the mid-1980s constitutes an exception. During the 1980s, male premature mortality was growing steadily in all CEE countries. A notable exception is Czechia, where a continuous decline in mortality was observed over the whole period. Poland and Slovakia joined this positive trend around 1990, which coincided with the collapse of the communist system in the CEE region. A few years later, Hungary also experienced the onset of a mortality reduction process. Finally, in the second half of the 1990s male mortality started to decline in Romania as well. By 2019, we observed comparable mortality levels for all countries of the CEE block except Czechia. Among all countries under consideration, Czechia is the vanguard in terms of the lowest male premature mortality.

### Spatial mortality patterns and clusters

**Figure 2:**
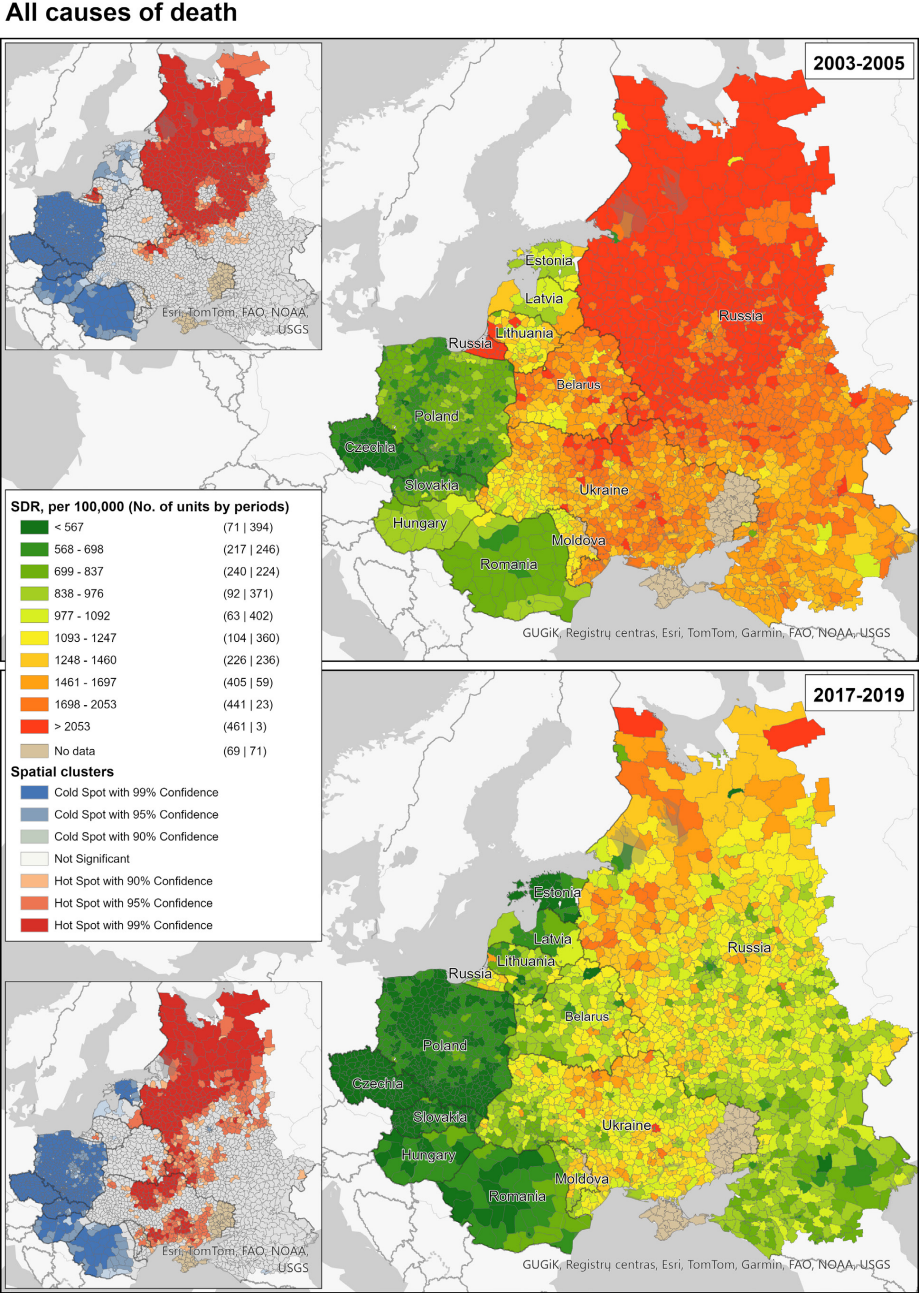
Spatial distribution of male premature mortality in 12 former Soviet Union and Central-Eastern Europe countries; all causes combined, 2003–2005 and 2017–2019. Source: own calculations based on harmonised regional mortality data. SDR, standardised mortality rate.

Figure 2 depicts the spatial patterns and clusters of male premature all-cause mortality in 12 CEE and FSU countries in 2003–2005 and 2017–2019. In 2003–2005, we can observe a clear geographical divide in terms of mortality levels between the CEE and FSU country blocks. Male premature mortality in the FSU countries was substantially higher. Clear discontinuities of spatial patterns existed along the Polish-Belarussian and Polish-Ukrainian national borders. On the other hand, similar mortality levels could be observed in border regions at the Hungarian-Ukrainian border and at the western part of the border between Ukraine and Romania. By 2017–2019, all countries and spatial units had experienced substantial reductions in male premature mortality levels. At the same time, the discontinuities of mortality patterns between the CEE and FSU country blocks at the national borders became more pronounced. Although the observed spatial patterns in 2017–2019 are more scattered compared with 2003–2005, large clusters of elevated and low male premature mortality can still be identified.

The spatial patterns of all-cause male premature mortality are largely driven by cardiovascular mortality and mortality from external causes (online supplemental figures S2 and S3). This can be inferred from both the very similar spatial patterns of mortality from these causes and their levels relative to all-cause mortality. This is not surprising given that cardiovascular mortality and mortality from external causes traditionally determine male premature mortality in the region. As with all-cause mortality, the geographical divide in external causes of death between the CEE and the FSU became more apparent in 2017–2019. In 2003–2005, mortality from external causes in western Ukraine, southwestern Belarus and Moldova was much closer to the level observed in the neighbouring CEE regions. Likewise, spatial patterns of both cardiovascular and external causes of mortality have become more scattered in the most recent years compared with the early 2000s. There are significant differences in mortality levels between the CEE and FSU countries, which is reflected by quite different SDR distributions across spatial units (online supplemental figure S4). This is particularly noticeable for the initial point of our analysis with the more stretched distribution for the FSU countries that is shifted to the right according to the level of observed mortality. By 2017–2019, the differences in mortality levels between the CEE and FSU countries became less pronounced, especially with respect to mortality with external causes of death. This was mainly achieved by a remarkable shift of the FSU distribution to the left (mortality reduction), which also became a way more homogenous distribution compared with the one observed in 2003–2005.

Nevertheless, it is clear that the CEE and FSU are very distinct in terms of mortality levels. Thus, our subsequent analyses are stratified by these two groups. The mortality maps, which served as the basis for the analysis of the hot spots and cold spots, appear in the online supplemental figures S5–S7.

**Figure 3:**
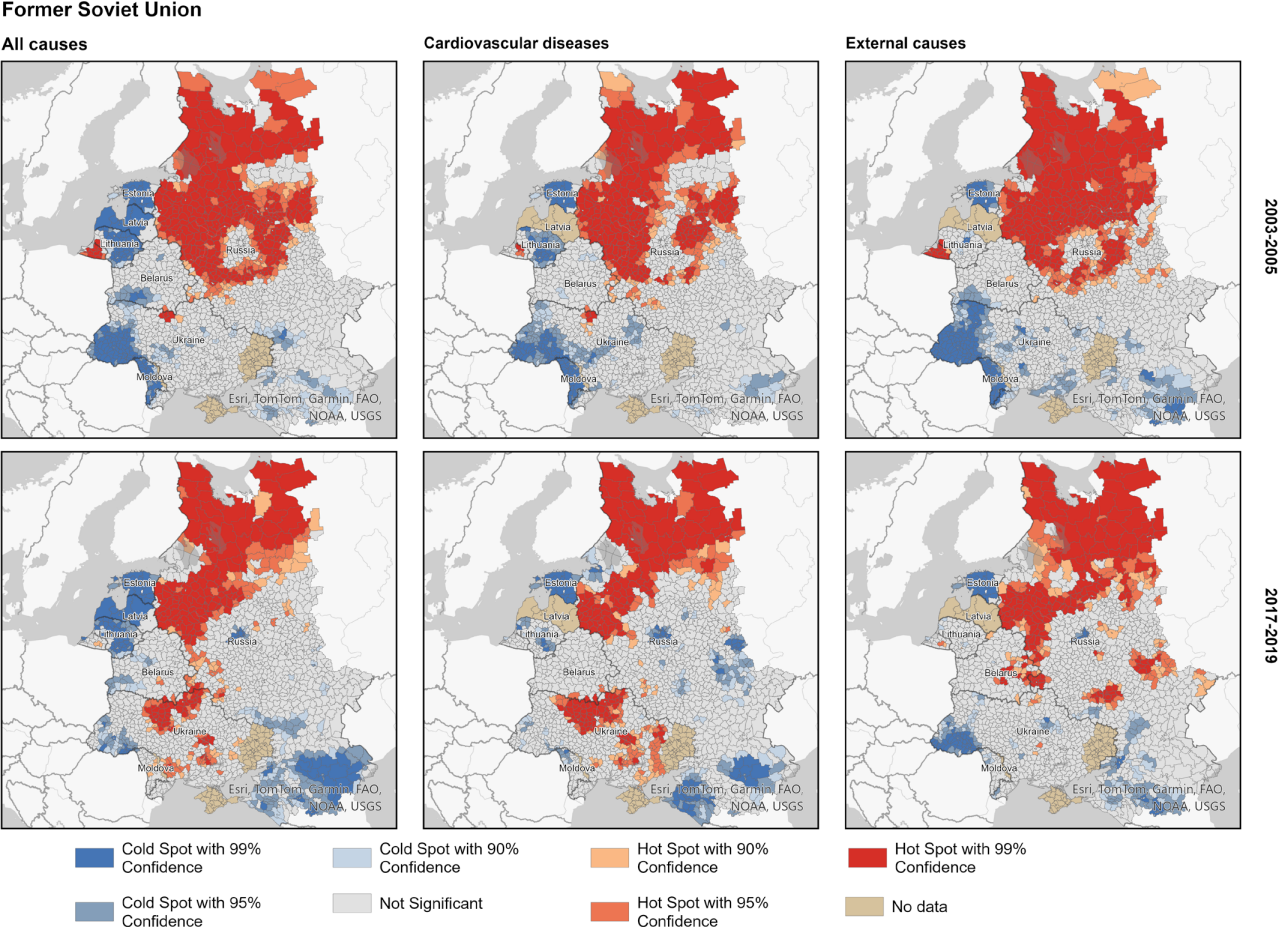
Hot and cold spots of male premature mortality within the former Soviet Union country block; major causes of death, 2003–2005 and 2017–2019. Source: as for figure 2.

Figure 3 exhibits for 2017–2019 geographical disparities in male premature mortality in each block of the countries. The spatial patterns for 2003–2005 reveal similar but somewhat larger clusters of both elevated and low mortality. Within the FSU country block, the hot spots of male premature mortality are predominantly located in the northwestern and western parts of European Russia. Hot spots can also be observed in northern Ukraine in the areas surrounding the capital, as well as in the northern areas contaminated due to the Chornobyl accident in 1986. The clusters of lowest mortality (cold spots) are located in the Baltic States, the southern part of European Russia, and to a lesser extent in southwestern Ukraine and Belarus. The spatial patterns of hot spots and cold spots for cardiovascular mortality and mortality from external causes look similar to those of all-cause mortality.

**Figure 4:**
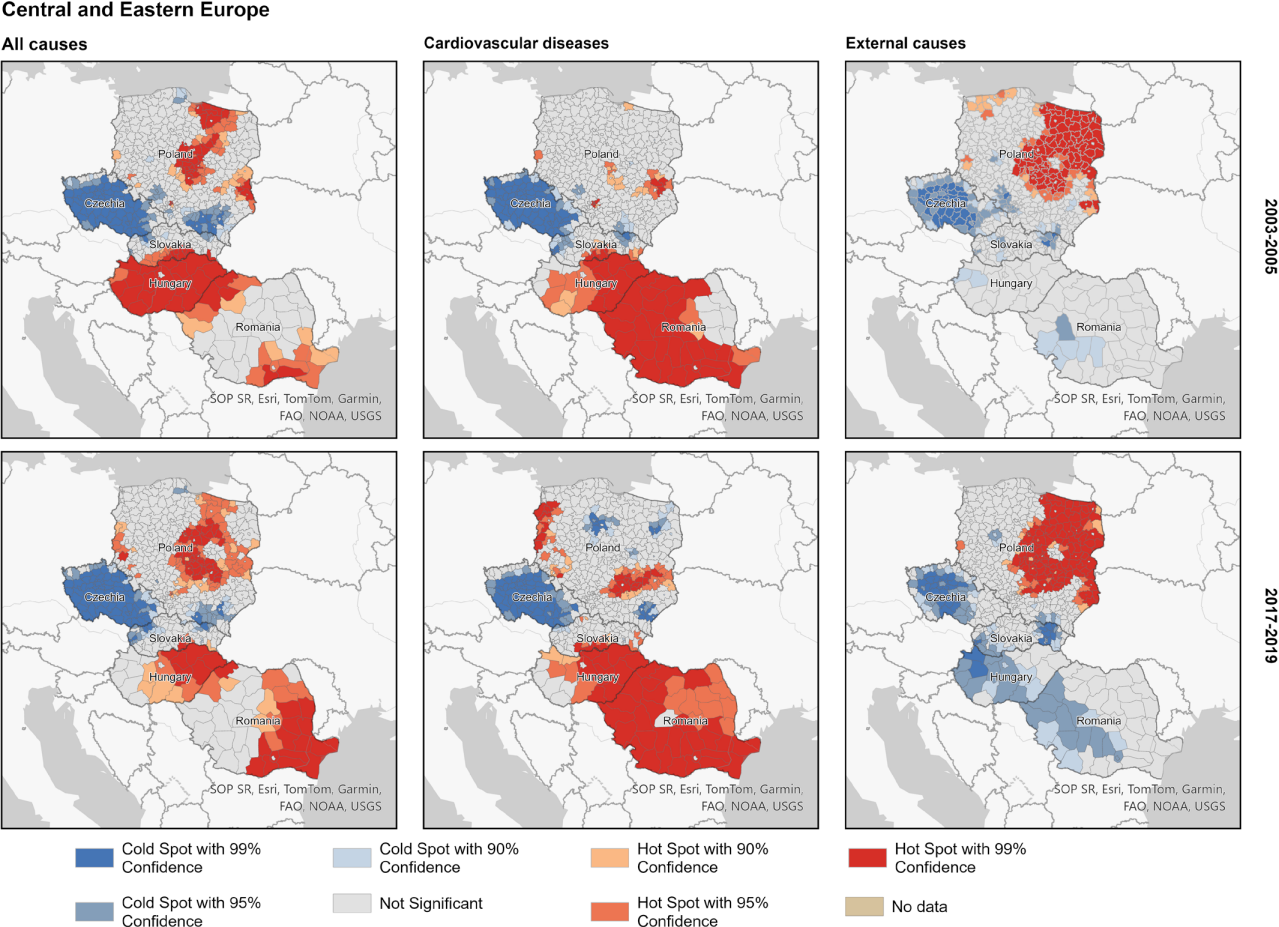
Hot and cold spots of male premature mortality within the Central-Eastern Europe country block; major causes of death, 2003–2005 and 2017–2019. Source: as for figure 2.

Within the CEE block of countries (figure 4), the majority of hot spots are located in Hungary (except for the western part), eastern Romania and the central and eastern parts of Poland. A few hot spots can also be identified in the northwestern regions of Romania at the border to Hungary, as well as in the regions of western Poland bordering eastern Germany. Unlike in the FSU countries, the spatial patterns of the leading causes of death (CVD and external) do not mirror those of all-cause mortality. The only exception is Czechia, which exhibits low mortality from both CVD and external causes of death. In Romania and Hungary, the vast majority of regions exhibit elevated CVD mortality, which is not the case as far as external causes of deaths are concerned. On the other hand, large cold-spot clusters of male premature mortality from external causes are located in these two countries. By contrast, almost all hot spots of mortality from this cause of death are located in the central part of Poland as well as the Polish regions along the borders with Belarus (east), Ukraine (southeast) and Russia (northeast).

### Assessing spatial inequalities

The observed changes in the spatial mortality patterns were accompanied by a substantial reduction in mortality across all countries and spatial units. Between 2003–2005 and 2017–2019, all-cause SDR declined on average from 1635 to 1005 deaths per 100 000 (table 1). The mortality reduction was particularly pronounced across the FSU districts, where SDR reduced by two thirds on average (1823 vs 1101). Meanwhile, the average SDR across the CEE districts declined by one third.

**Table 1:** Descriptive statistics and dispersion measures calculated across spatial units of FSU and CEE countries in 2003–2005 and 2017–2019

	All countries		FSU		CEE	
	2003–2005	2017–2019	2003–2005	2017–2019	2003–2005	2017–2019
Mean	1635	1005	1823	1101	705	529
Median	1668	1024	1783	1085	706	532
SD	618	337	498	282	117	99
Theil index	0.076	0.058	0.038	0.033	0.014	0.018
Between	0.046	0.029	0.006	0.002	0.005	0.008
Within	0.030	0.029	0.032	0.031	0.009	0.010
N	2320		1723		597	

Source: own calculations based on the age-standardised death rates from all causes combined per 100 000 population.

CEE, Central-Eastern Europe; FSU, former Soviet Union.

The Theil index indicates an overall reduction of the degree of heterogeneity across all spatial units: 0.076 in 2003–2005 versus 0.058 in 2017–2019. It is important to note that by the end of the observation period, both ‘between’ and ‘within’ components contribute equally to the total value of the Theil index. This implies an equal relevance of mortality variation between countries compared with mortality variation within countries in terms of the contribution to the total variation observed across all spatial units. By contrast, in 2003–2005, the ‘between’ component played a more prominent role. With this respect, the block of the FSU countries is an exception: the ‘within’ component determines the Theil index while the contribution of the ‘between’ component was small in 2003–2005 and has become negligible in 2017–2019.

## Discussion

### Main findings

This study provides the first comprehensive and detailed evidence of changing spatial patterns in male premature mortality across small territorial units in the CEE–FSU region from the early 2000s to the late 2010s. Identifying these spatial patterns provides important insights into the progress in combating the consequences of the massive mortality crisis that emerged with the collapse of the communist rule. The findings also highlight newly emerged mortality divides within the region and within single countries. The analyses of persistence and change in these spatial patterns with a focus on cross-border regions are highly relevant for identifying persistence of the past and newly emerging spatial disparities.

Several conclusions can be drawn from the analysis of spatial patterns of all-cause premature mortality. First, a notable contrast in levels of male premature mortality between the EU member countries and Russia, Belarus, Ukraine and Moldova observed in the first half of the 2000s persisted in the second half of the 2010s. Thus, the lack of convergence at the national level in these laggard countries towards the CEE and Baltic countries can be partially explained by highly variable progress and stalling improvements in numerous disadvantaged regions. Both diseases of the cardiovascular system and external causes of death contribute to this persisting geographical pattern. An exception is several areas in Lithuania, which show higher mortality due to these causes of death than neighbouring Polish, other CEE and Estonian regions. Second, our analyses also revealed significant differences within (a) The group of FSU countries including the Baltic States and (b) The group of CEE countries, respectively. Within the FSU block, one of the most pronounced and systematic divides persists between Russia and the Baltic States, showing high excess male premature mortality due to cardiovascular system diseases and external causes of death in the neighbouring Russian border regions. Another significant divide within the FSU can be observed between Ukraine and Belarus, with the neighbouring regions in northern Ukraine showing higher mortality due to CVDs. Interestingly, similar sharp divides are also evident within CEE countries having substantially lower mortality levels. For example, a high mortality hot spot attributable to excess cardiovascular mortality in eastern Hungary contrasts with much lower mortality in the neighbouring Slovakian regions. A large cross-border area between Hungary and Romania forms a high male premature mortality zone attributable to excess cardiovascular mortality. Third, important differences also persist within single countries. The most pronounced and systematic gradients include (a) A North-West versus South gradient in Russia, (b) East–West mortality divides in Hungary, Romania and Poland and (c) North–East versus West mortality gradient in Ukraine.

### Interpretation

Distinctive cause-specific patterns behind the hot and cold spots of total male premature mortality highlight the role of specific determinants. First, the role of violent deaths was much more important for male premature mortality in the group of former USSR countries, with northwestern Russia constituting a hot spot of exceptionally high mortality. Although at the lower mortality level, a similar hotspot in CEE was found in eastern parts of Poland, Hungary and Romania. Prior studies highlight strong relationships between excess male premature mortality, violence and prevailing harmful alcohol consumption patterns in the former USSR countries.^21^,^23^ It seems that the modern geographical patterns of alcohol-related and violent mortality may at least be partially attributed to a historical legacy and its persistence. For example, high alcohol-related mortality can be observed in the European part of the Russian Empire (also including the modern Baltic States and eastern Poland) already at the end of the 19th century, with the ethnically Russian provinces showing more unfavourable rates than other provinces (eg, Belarussian, Ukrainian and Baltic provinces).^12^ It has also been shown that this historical pattern became even more pronounced during the period of communist rule and the transitional period of the 1990s, and prevailed during the 2000s and early 2010s.^7 12^ Exceptionally high alcohol-related mortality persisted in specific areal clusters such as northwestern Russia (except St. Petersburg and Moscow regions).^7^ During the period of communist rule, these specific areas (typically rural areas or industrial towns) were hit hard by shortcomings of the Soviet centrally planned economy, leading to socioeconomic stagnation, poor living conditions, limited future prospects, selective outmigration and depopulation.^24 25^

The persisting geographical patterns and, especially, pronounced hot and cold spots suggest still modest and fragmented impacts of the most recent political, socioeconomic and health system transformations in the region. It has been suggested that even at the national level, the 2004 EU enlargement was not followed by systematic and uniform convergence in life expectancy between new and old member states.^1 26 27^ For example, no clear signs of mortality convergence were observed across the Polish and Czech regions despite these countries showing the region’s highest and most improved national life expectancies.^26^ It is possible that the 2004 EU enlargement effect is hard to detect either due to the potential positive influence of pre-accession processes (ie, aligning policies and implementing reforms) or because it requires a much longer time to affect mortality indicators.^26^ In addition, despite substantial structural financial support to the new EU member states, the EU has maintained little role in transforming healthcare and social systems.^27^ Therefore, the policy-driven health progress in the new EU member states has been largely driven by national priorities (reforms and investments) and specific policy actions.

The selected non-EU countries, such as Russia, Belarus, Ukraine and Moldova, show even larger diversity in the political and socioeconomic contexts. For example, the pronounced economic growth of Russia attributable to spiking oil and gas prices after 2000 was accompanied by rapid reductions in national mortality since the mid-2000s.^28^ The rapid growth in national income during the 2000s allowed the improvement of socioeconomic conditions and massive investments in the health system infrastructure and national health programmes.^29^ However, as our study and other evidence also confirm, the observed mortality reductions in Russia were highly uneven, with the initially worst regions lagging without any signs of systematic convergence towards the best-performing richest regions of Moscow and St. Petersburg.^9 29^ Unlike Russia, the countries Belarus, Ukraine and Moldova did not maintain such resources and could not implement similar-scale investments in healthcare infrastructure. Although Ukraine also experienced a notable increase in life expectancy since 2008 and initiated healthcare reforms in 2015–2017, this positive trend was interrupted in 2014 and stalled after 2017 due to numerous obstacles, including the onset of war in Donbas and Russia’s annexation of Crimea. As evidence from Russia, Belarus and Lithuania shows, recent implementations of strong health policy measures (especially alcohol control policies) and prevention may also be very effective for achieving mortality reductions, especially in the case of premature mortality.^4 28 30^ However, although these policies were associated with progress at the national level, they did not eliminate pronounced alcohol-related mortality hot spots in the region.^4^ Further lowering external mortality among men also depends on the progress in reducing suicide deaths through policies addressing mental health problems. This is particularly relevant for such countries as Lithuania, Estonia, Russia and Hungary.^31^ Persisting inequalities between and within the EU and non-EU countries of the CEE–FSU region can also be associated with persisting structural divides in education, employment structure and many other characteristics.^32^

### Strengths and limitations

Our analyses rely on the unique collection of the regional cause-specific mortality data. Whereas such data are generally accessible for the most recent years and the CEE countries, it took us a tremendous amount of effort to obtain the data for the FSU countries, especially for the earlier periods. Another challenge was to harmonise the data and ensure their comparability within and between countries as well as over time. This included the adjustments for territorial changes according to the most recent administrative division, grouping deaths into broad groups of causes and redistributing ill-defined causes of death within each population strata. Limiting the analysis to ages 20–64 was partly motivated by higher quality of cause-of-death registration at these ages compared with older ages. The key strength of our analyses is the ability to offer a broad spatial perspective and assess for the first time the temporal changes in the spatial patterns of male premature mortality across a large number of CEE–FSU countries. Nevertheless, few limitations have to be mentioned. First, due to data availability issues, our analyses do not cover the 1990s, the period where male premature mortality exhibited particularly in Eastern Europe the most dramatic changes as highlighted by figure 1. However, focusing on the period since the early 2000s, which was partly driven by data availability and data comparability considerations (cause-specific mortality data for all countries are based on one classification, the ICD–10), has its advantages. In the period between 2003–2005 and 2017–2019, male premature mortality was quite steadily declining in all countries, which allowed us to pool years together and ensure more robust mortality rates. Averaging over several years would be problematic and less meaningful for the 1990s, where mortality changed so abruptly from 1 year to another. The second limitation concerns the comparability of the analysed periods between countries at the initial point of our analysis, 2003–2005. Due to the data constraints, we had to use the data for adjacent periods for Moldova, Russia and Ukraine. Nevertheless, these few mismatches should not have a significant impact on our conclusions, as the mortality levels observed in these countries (see figure 1) were rather stable and quite comparable between the alternative periods. Finally, our findings may be influenced by the modifiable areal unit problem, whereby mortality estimates and their interpretations can vary depending on the scale and definition of the geographic units used.^33^

## Supplementary material

10.1136/bmjph-2025-003714 online supplemental file 1

## Data Availability

Data may be obtained from a third party and are not publicly available.
