# Supplementary material for: Geographical divides in male premature mortality in the CEE–FSU European region: an ecological study of 2320 spatial units in 12 countries, 2003–2019
Source: BMJ Public Health. 2026 Feb 20;4(1):e003714. doi: 10.1136/bmjph-2025-003714 (PMC12927323; doi:10.1136/bmjph-2025-003714)
Supplement: online supplemental file 1 [file bmjph-4-1-s001.pdf]

# ONLINE SUPLEMENTARY FOR

## Geographical divides in male premature mortality in the CEE–FSU European region: an ecological study of 2320 spatial units in 12 countries, 2003–2019

Pavel Grigoriev[1*], Domantas Jasilionis[2,3], Sergey Timonin[4,5], Nataliia Levchuk[2,6], Pavlo Shevchuk[6], Olga Penina[7], Katalin Kovács[8], Sebastian Klüsener[1,3,9]

[1] Federal Institute for Population Research (BiB), Wiesbaden, Germany

[2] Max Planck Institute for Demographic Research, Rostock, Germany

[3] Vytautas Kavolis Transdisciplinary Research Institute, Vytautas Magnus University, Kaunas, Lithuania

[4] School of Demography, Research School of Social Sciences, The Australian National University, Canberra, Australia;

[5] Centre of Epidemiology for Policy and Practice, National Centre for Epidemiology and Population Health, The Australian National University, Canberra, Australia.

[6] Mykhailo Ptukha Institute for Demography and Life Quality Research, Kyiv, Ukraine

[7] Nicolae Testemitanu State University of Medicine and Pharmacy, Chisinau, Moldova

[8] Hungarian Demographic Research Institute, Budapest, Hungary

[9] Department of Sociology and Social Psychology (DSS), University of Cologne, Cologne, Germany

***Correspondence to**: Pavel Grigoriev (Federal Institute for Population Research (BiB), Friedrich-Ebert-Allee 4, 65185 Wiesbaden, Germany. Email: Pavel.Grigoriev@bib.bund.de ).

## A. Methods: Theil Index and its decomposition

## B. Supplementary Tables

Table S1. Description and format of the available raw regional mortality data in 12 CEE and FSU countries

## B. Supplementary Figures

Figure S1. Study area of the analysis of male premature mortality in Central and Eastern Europe

Figure S2. Spatial distribution of male premature mortality in 12 FSU and CEE countries; cardiovascular diseases, 2003–2005 and 2017–2019

Figure S3. Spatial distribution of male premature mortality in 12 FSU and CEE and countries; external causes, 2003–2005 and 2017–2019

Figure S4. Distribution of the observed value of the standardized death rate by spatial units of the FSU and CEE countries, 2003–2005 and 2017–2019

Figure S5. Spatial distribution of male premature mortality within the CEE and FSU country blocks; all causes combined, 2003–2005 and 2017–2019

Figure S6. Spatial distribution of male premature mortality within the CEE and FSU country blocks; cardiovascular diseases, 2003–2005 and 2017–2019

Figure S7. Spatial distribution of male premature mortality within the CEE and FSU country blocks; external causes, 2003–2005 and 2017–2019

## A. Methods: Theil Index and its decomposition

To measure mortality inequalities across countries and spatial units, we used the **Theil Index (T)**, an entropy-based measure of inequality. An important feature of the Theil Index is that it can be decomposed additively into **between-country ($T_B$) and within-country ( $T_W$ )** components. This decomposition allows the distinction of inequality attributable to differences *between countries* versus *differences among regions within countries.*

The **overall Theil Index** is defined as:

$$T = \frac{1}{N}\sum_{i=1}^{c}\sum_{j=1}^{n_i}\frac{SDR_{ij}}{\overline{SDR}}\,ln\frac{SDR_{ij}}{\overline{SDR}}$$

where: $SDR_{ij}$ is our mortality measure, the standardized death rate (SDR) in region *j* of country *i.*

$\overline{SDR}$ is the global mean across all regions in all countries defined as:

$$\overline{SDR} = \frac{1}{N}\sum_{i=1}^{c}\sum_{j=1}^{n_i} SDR_{ij}$$

$N$ is the total number of regions across all countries.

$n_i$ is the number of regions in country *i.*

$c$ is the total number of countries.

The **between-country Theil Index** representing the fraction of total inequality due to differences between countries is defined as:

$$T_B = \frac{1}{N}\sum_{i=1}^{c} n_i\frac{\overline{SDR_i}}{\overline{SDR}}\,ln\frac{\overline{SDR_i}}{\overline{SDR}}$$

The **within-country Theil Index** representing the fraction due to **differences among regions within countries** is defined as:

$$T_W = \frac{1}{N}\sum_{i=1}^{c}\sum_{j=1}^{n_i}\frac{SDR_{ij}}{\overline{SDR}}\,ln\frac{SDR_{ij}}{\overline{SDR_\iota}}$$

$$T = T_B + T_W$$

The **Theil Index ( $T$ )** is an additively decomposable measure. It can be easily decomposed into country-specific contributions and further dimensions of interest.

**Table S1. Description and format of the available raw regional mortality data in 12 CEE and FSU countries**

| Country | Years | | Number of spatial units | Available classification of causes of death | Comments |
|---|---|---|---|---|---|
| | available | used in the analysis | | | |
| Central and Eastern Europe (CEE) | | | | | |
| Czechia | 1990–2019 | 2003–2005, 2017–2019 | 77 | Full ICD–9/10 List | |
| Hungary | 1991–2019 | | 20 | SIM List[1] | Age-specific mortality rates |
| Poland | 2002–2020 | | 379 | Full ICD–10 List | |
| Romania | 1996–2020 | | 42 | 18 broad groups | |
| Slovakia | 1996–2020 | | 79 | Full ICD–9/10 List | |
| CEE | | | 597 | | |
| Former Soviet Union (FSU) | | | | | |
| Belarus | 2003–2019 | 2003–2005, 2017–2019 | 127 | Country-specific abridged classification (<300 items) based on the ICD–10 | |
| Estonia | 2000–2019 | | 15 | SIM List* | Age-standardized death rates |
| Latvia | 1996–2021 | | 12 | - | No cause-specific data |
| Lithuania | 2001–2020 | | 60 | Full ICD–9/10 List | |
| Moldova | 1991–2020 | 2002–2006, 2017–2019 | 30 | Full ICD–9/10 List | 1.Mortality rates for 2002–2006 are centered around the 2004 Population Census 2.No data for Transnistria[2] (N=1) |
| Russia (European part) | 2000–2020 | 2001–2003, 2017–2019 | 1008 | Full ICD–9/10 List | 1.Mortality rates for 2001–2003 are centered around the 2002 Population Census 2.No data for two units in Moscow oblast in 2017–2019 |
| Ukraine | 2006–2019 | 2006–2008, 2017–2019 | 471 | Country-specific abridged classification (<270 items) based on the ICD–10 | No data for Crimea[3], Donetsk, and Luhansk oblast (N=68) |
| FSU | | | 1723 | | |
| FSU+CEE | | | 2320 | | |

Notes: 1) The so-called short-intermediate (SIM) list containing 57 items was developed in the framework of a project aimed at restoring a long retrospective cause-of-death time series. The groups were produced as the most appropriate solution to ensure comparability of major causes across several revisions of the International Classification of Diseases (ICD). 2) Transnistria is a breakaway region of Moldova which independence is not internationally recognized. 3) Crimea is internationally recognized as part of Ukraine but de facto controlled by Russia since 2014.

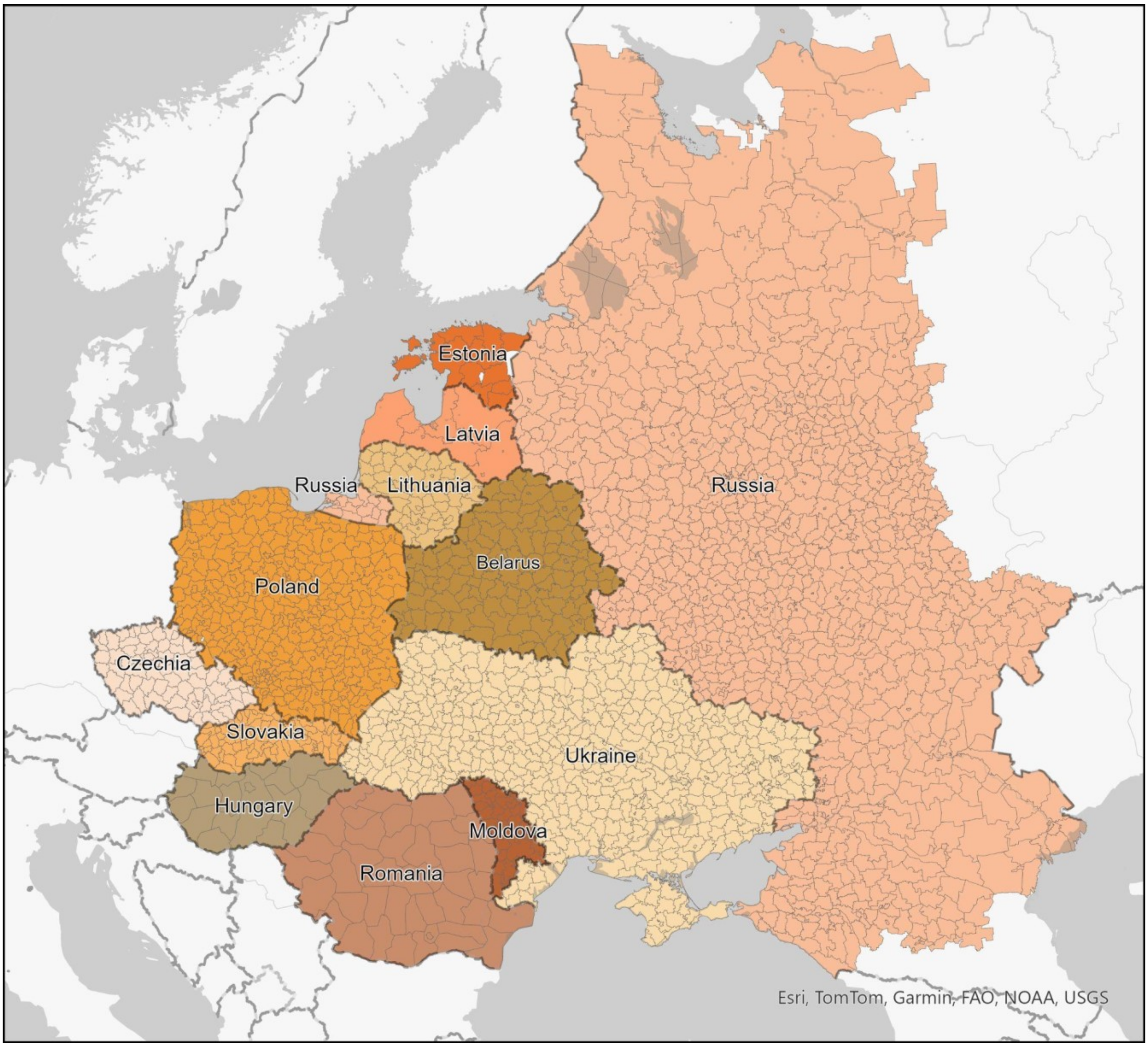


**Figure S1. Study area of the analysis of male premature mortality in Central and Eastern Europe**

Source: Prepared by the authors

# Cardiovascular diseases

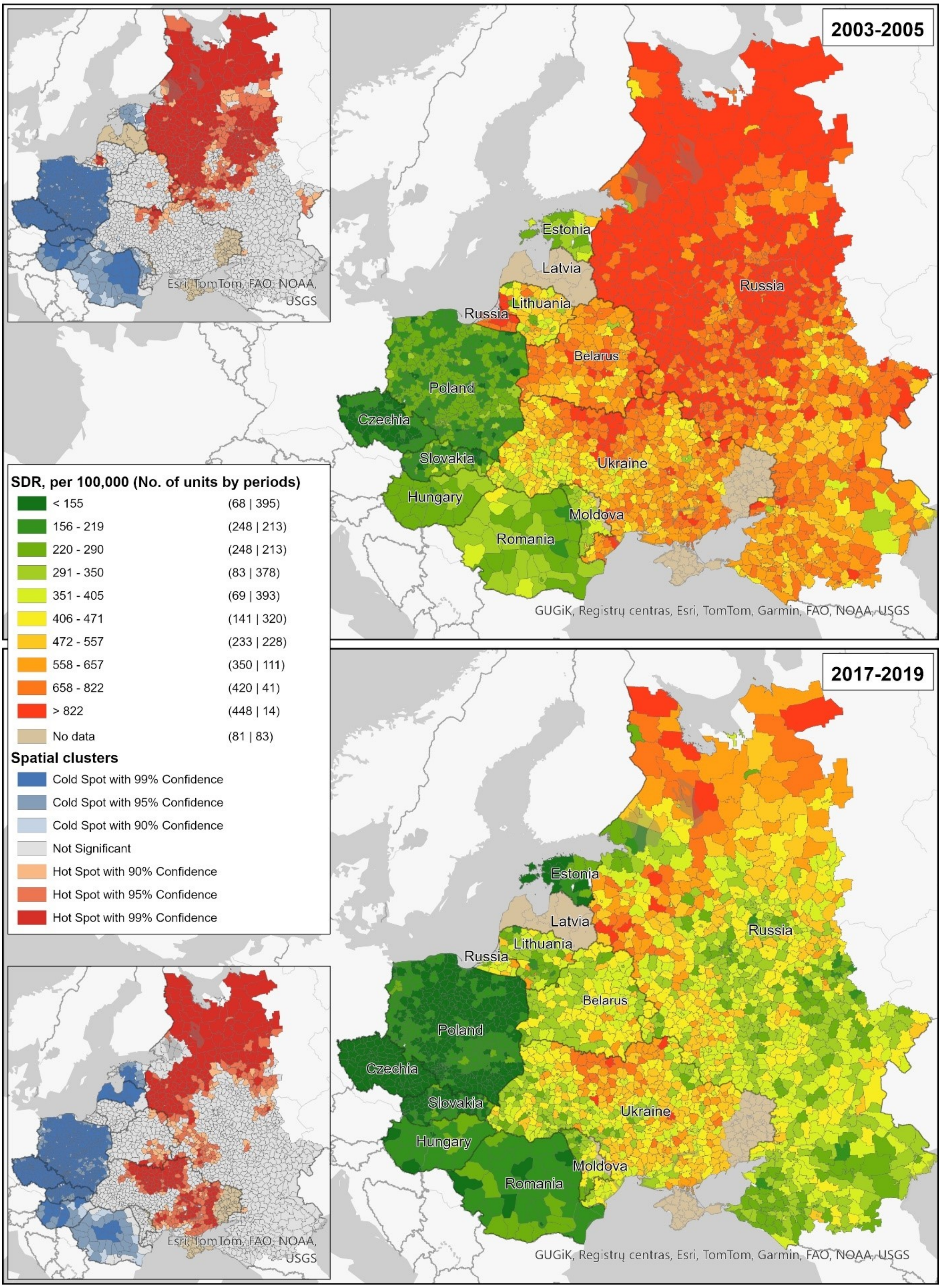


**Figure S2. Spatial distribution of male premature mortality in 12 FSU and CEE countries; cardiovascular diseases, 2003–2005 and 2017–2019**

Source: Own calculations based on harmonized regional mortality data

# External causes of death

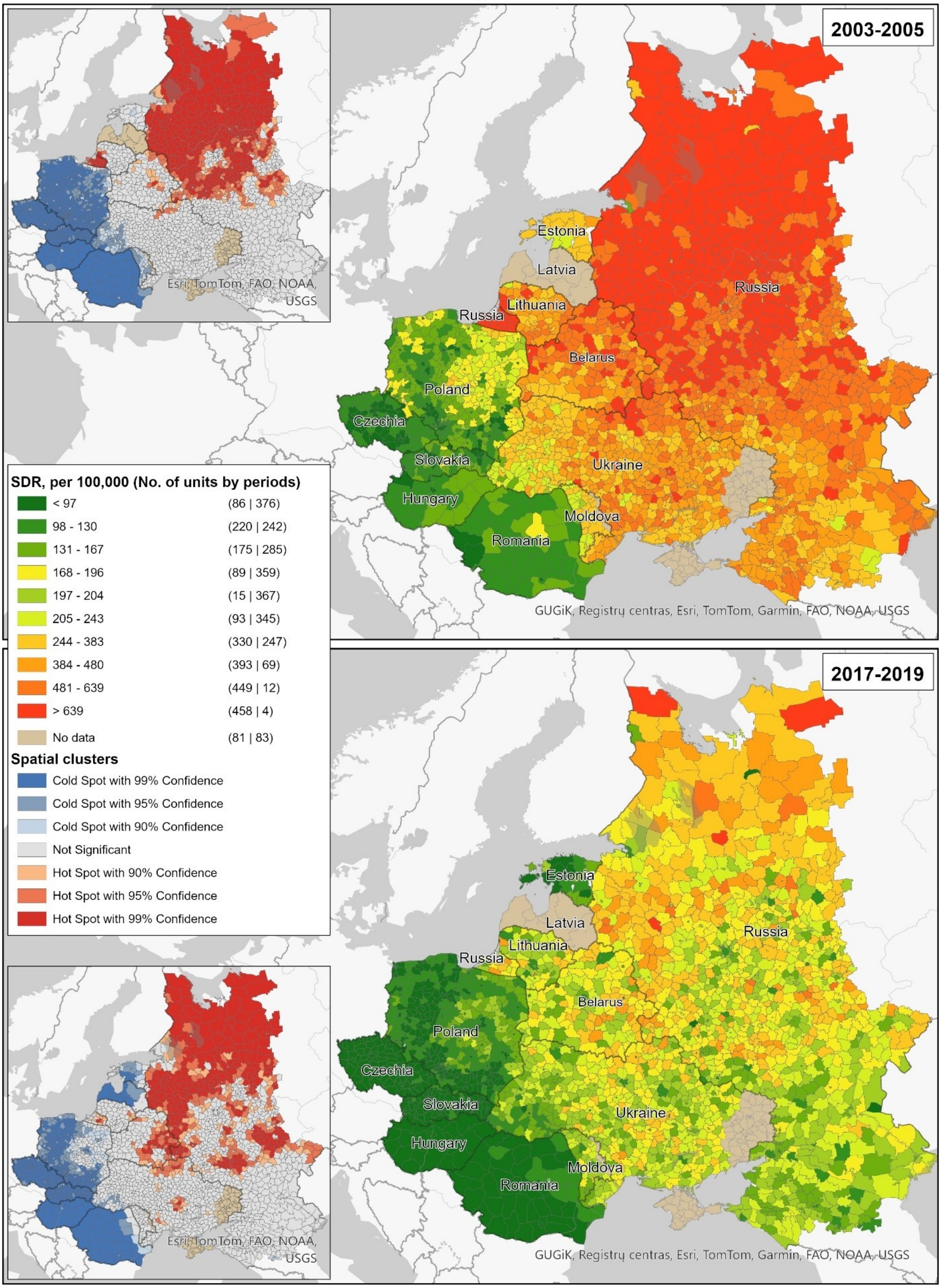


**Figure S3. Spatial distribution of male premature mortality in 12 FSU and CEE and countries; external causes, 2003–2005 and 2017–2019**

Source: As for Figure S2

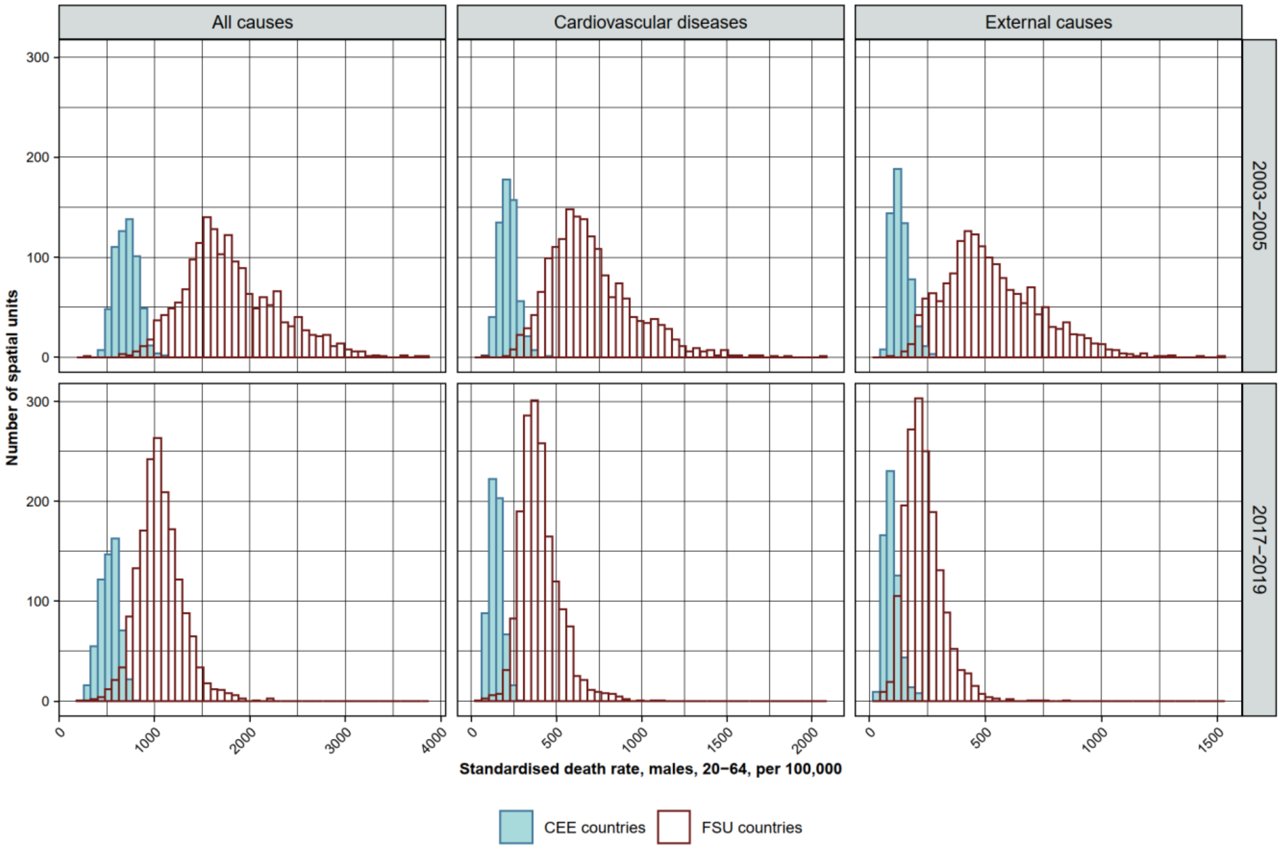


**Figure S4. Distribution of the observed value of the standardized death rate by spatial units of the FSU and CEE countries, 2003–2005 and 2017–2019**

Source: As for Figure S2

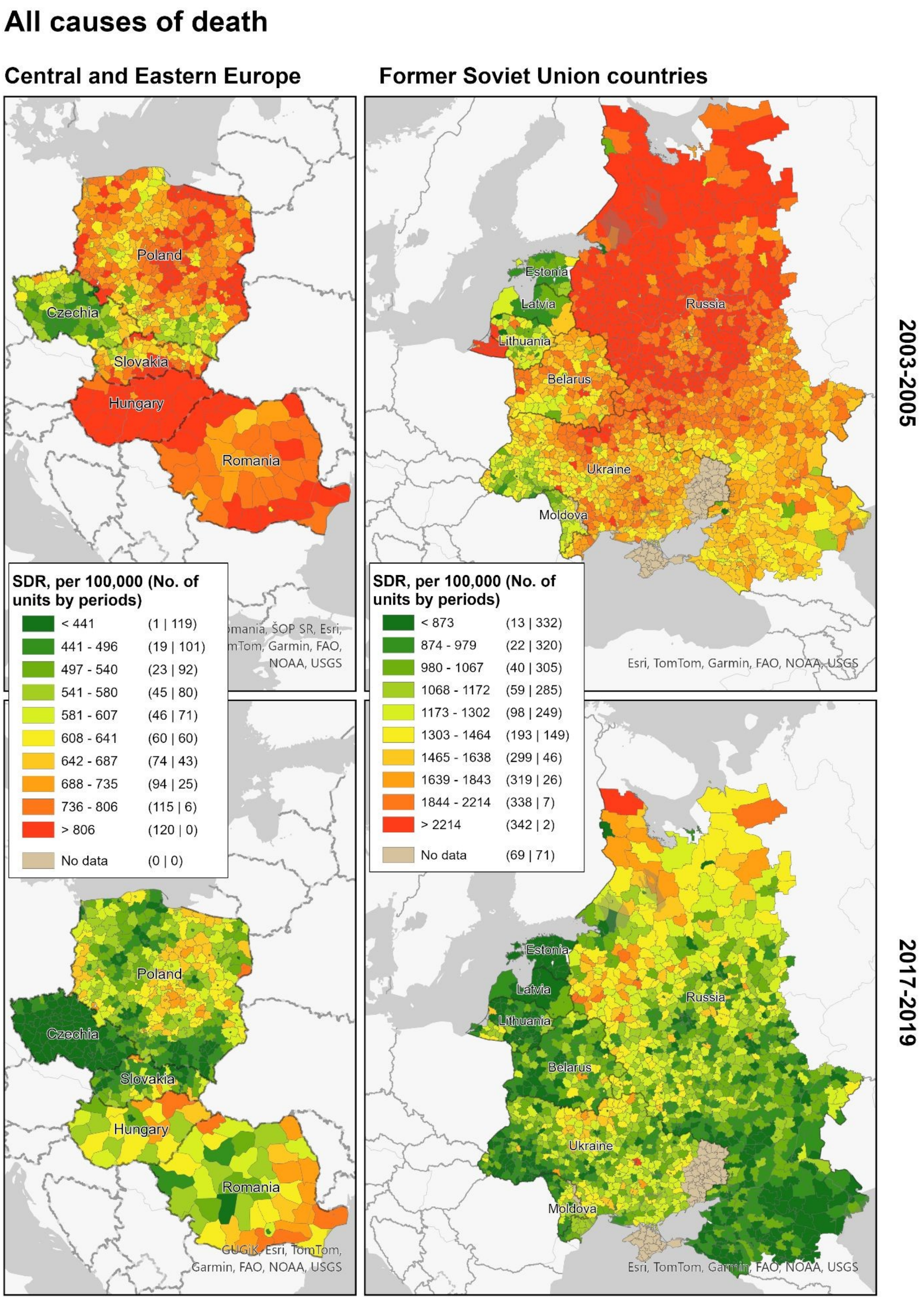


**Figure S5. Spatial distribution of male premature mortality within the CEE and FSU country blocks; all causes combined, 2003–2005 and 2017–2019**

Source: As for Figure S2

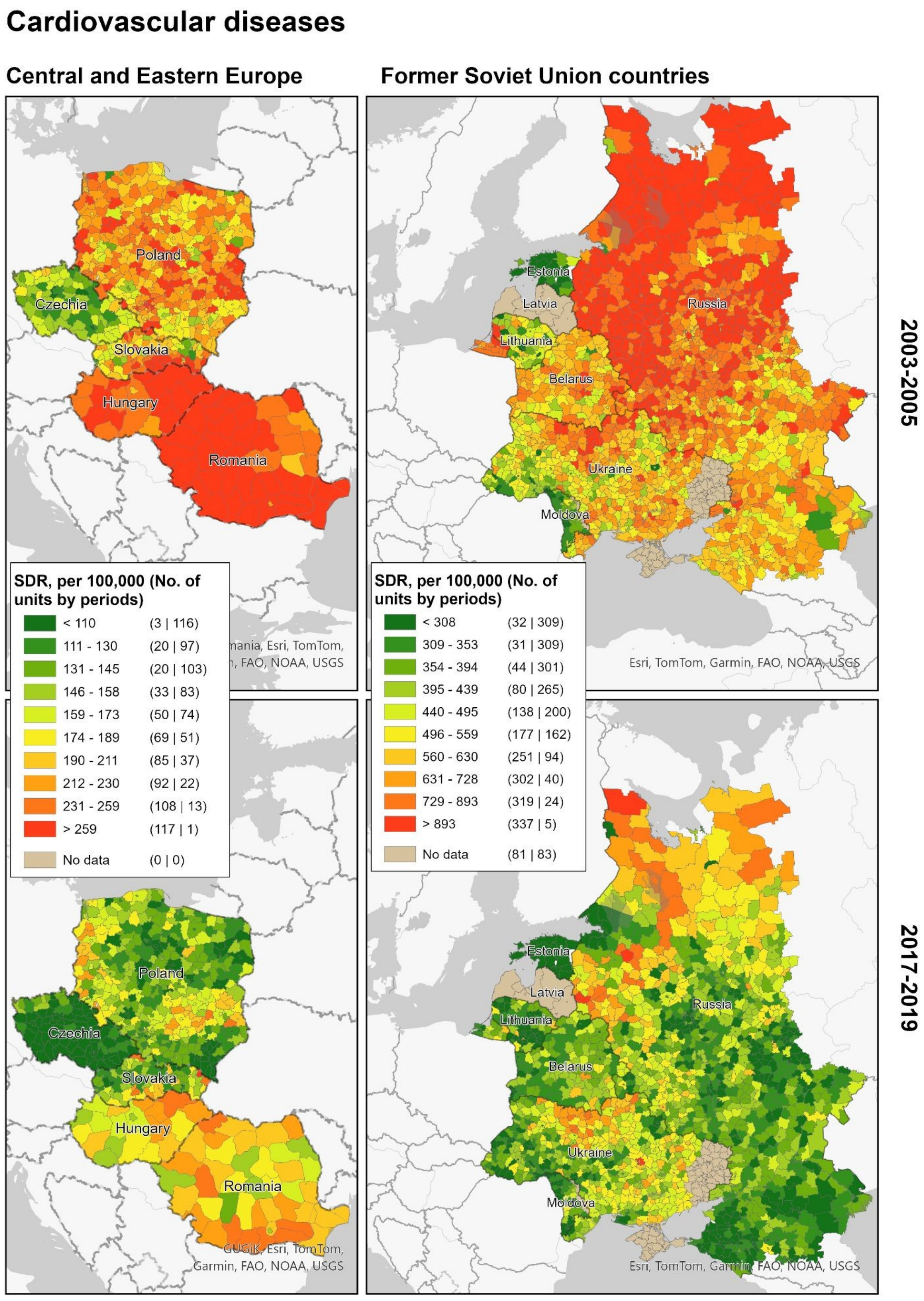


**Figure S6. Spatial distribution of male premature mortality within the CEE and FSU country blocks; cardiovascular diseases, 2003–2005 and 2017–2019**

Source: As for Figure S2

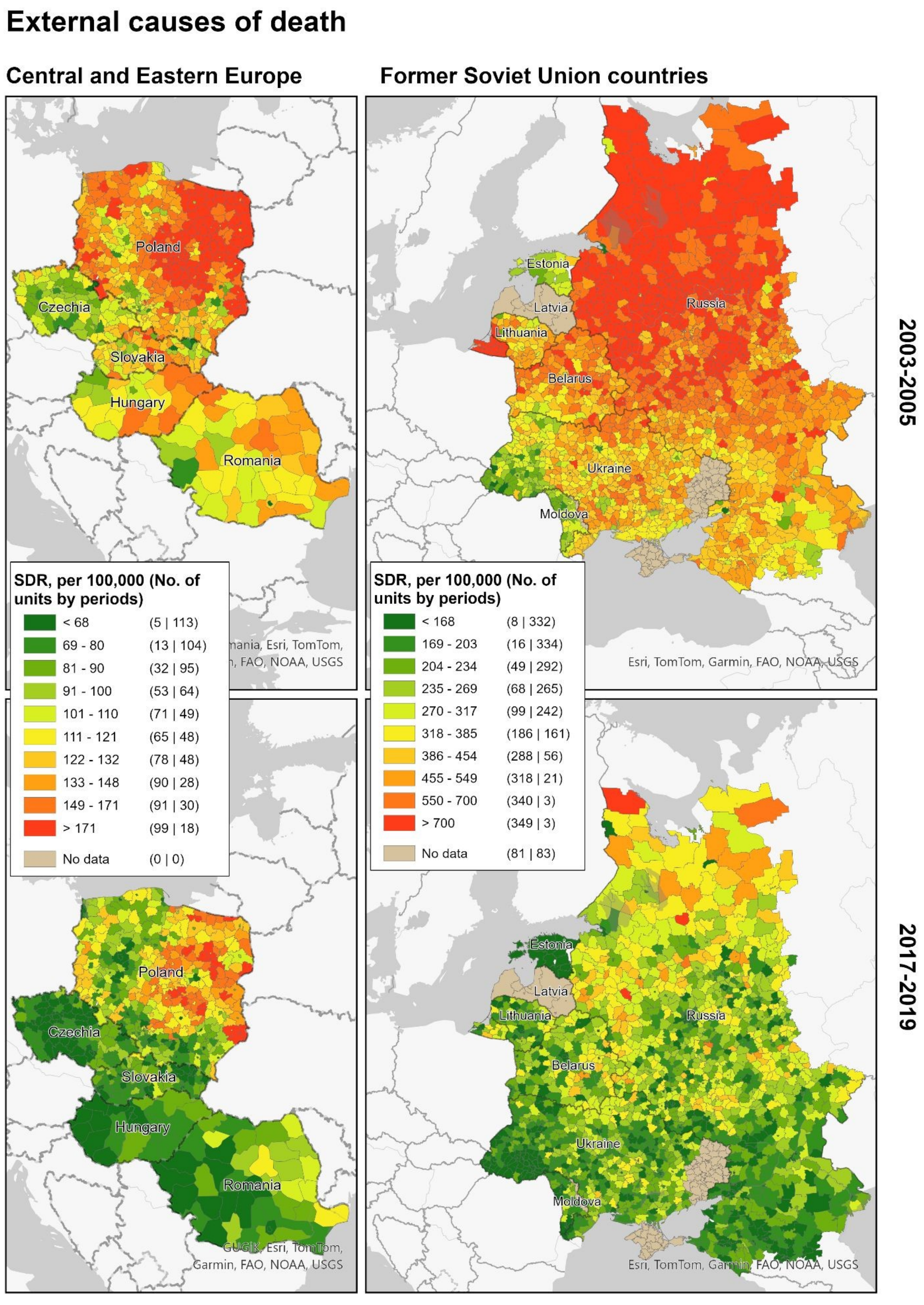


**Figure S7. Spatial distribution of male premature mortality within the CEE and FSU country blocks; external causes, 2003–2005 and 2017–2019**

Source: As for Figure S2
